# Epigenetic and biomolecular mechanisms in chronic temporomandibular disorder–related pain: an integrative review

**DOI:** 10.1007/s11033-026-11739-5

**Published:** 2026-04-02

**Authors:** Laís Valencise Magri, Melissa de Oliveira Melchior, Victor Hugo Alves Ribeiro-Silva, Jardel Francisco Mazzi-Chaves, Kranya Victoria Díaz-Serrano, Edilaine Cristina Silva Gherardi-Donato, Christie Ramos Andrade Leite-Panissi

**Affiliations:** 1 Departamento de Odontologia Restauradora, Faculdade de Odontologia de Ribeirão Preto, Universidade de São Paulo, Avenida do Café s/n, Monte Alegre, Ribeirão Preto, São Paulo, 14040-904 Brazil; 2 Departamento de Clínica Infantil, Faculdade de Odontologia de Ribeirão Preto, Universidade de São Paulo, Ribeirão Preto, São Paulo, Brazil; 3 Departamento de Enfermagem Psiquiátrica, Escola de Enfermagem de Ribeirão Preto, PAHO/WHO Collaborating Centre for Nursing Research Development, Universidade de São Paulo, Ribeirão Preto, São Paulo, Brazil; 4 Departamento de Psicologia, Faculdade de Filosofia Ciências e Letras de Ribeirão Preto, Universidade de São Paulo, Ribeirão Preto, São Paulo, Brasil

**Keywords:** Temporomandibular disorders, Chronic orofacial pain, Biomarkers, Epigenetics, Neuroendocrine regulation, Inflammation, Oxidative stress, Personalized medicine, Salivary diagnostics, Sex differences

## Abstract

Temporomandibular disorders (TMD) are multifactorial chronic pain conditions involving the temporomandibular joint, masticatory muscles, and associated structures, with a marked predominance in women. Despite their high prevalence and significant impact on quality of life, the biological mechanisms underlying pain chronification in TMD remain incompletely understood. Growing evidence indicates that persistent TMD-related pain arises from complex interactions among inflammatory signaling, oxidative stress, neuroendocrine dysregulation, and epigenetic modulation of gene expression. This integrative narrative review synthesizes current clinical and preclinical evidence from molecular biology, neuroendocrinology, and epigenetics to elucidate the biomolecular mechanisms involved in chronic TMD pain. Studies consistently report elevated proinflammatory cytokines, such as interleukin-6 and tumor necrosis factor-α, alongside increased oxidative stress markers, including malondialdehyde and 8-hydroxy-2′-deoxyguanosine, accompanied by reduced antioxidant capacity in saliva and serum. Alterations in neuroendocrine mediators, particularly dysregulation of the hypothalamic–pituitary–adrenal axis and reduced levels of neurotrophic factors such as brain-derived neurotrophic factor and nerve growth factor, appear to contribute to central sensitization and impaired neuroplasticity. In parallel, epigenetic mechanisms—including DNA methylation of pain- and stress-related genes (e.g., *COMT* and *TNFα*) and differential expression of microRNAs such as miR-140-5p and miR-21-5p—emerge as key modulators of pain susceptibility, persistence, and sex-related differences in clinical presentation. By integrating biomolecular, neuroendocrine, and epigenetic evidence, this review highlights biological signatures associated with chronic TMD pain and discusses their relevance for mechanistic understanding, biomarker discovery, and the development of biologically informed strategies for individualized pain management.

## Introduction

Temporomandibular disorders (TMD comprise a heterogeneous group of musculoskeletal conditions that affect the temporomandibular joint (TMJ), masticatory muscles, and surrounding tissues, frequently characterized by chronic orofacial pain, limited mandibular function, and a significant psychosocial burden. Despite their high prevalence—particularly among women—and their recognized biopsychosocial impact, the mechanisms underlying the chronification of TMD pain remain only partially understood. Recent advances in molecular, neuroendocrine, and epigenetic research suggest that chronic TMD pain arises from complex biological dysregulation involving inflammation, oxidative stress, hormonal modulation, and alterations in gene expression [1, 6]. Despite the scientific maturity of these findings, they remain underutilized in clinical practice. To date, no review has systematically integrated these biomolecular mechanisms into a translational and clinically actionable framework for diagnosing and managing TMD. This review addresses the critical gap by synthesizing current evidence and proposing strategies for applying salivary and serum biomarkers in precision-oriented orofacial pain care [7, 12].

Mounting data indicate that chronic inflammation is a core feature of TMD-related pain. Elevated levels of proinflammatory cytokines, including interleukin-6 (IL-6), tumor necrosis factor-alpha (TNF-α), and interleukin-1 beta (IL-1β), have been identified in the synovial fluid, saliva, and serum of individuals with chronic TMD [3, 6, 8, 10, 13, 14]. These mediators not only perpetuate peripheral sensitization but also interact with molecular pathways that promote central sensitization and neural plasticity. Simultaneously, oxidative stress plays a critical role in sustaining the inflammatory environment. Biomarkers such as malondialdehyde (MDA), 8-hydroxydeoxyguanosine (8-OHdG), and total lipid hydroperoxides are consistently elevated in both plasma and saliva of TMD patients, indicating a persistent imbalance between the production of reactive oxygen species (ROS) and antioxidant defense systems [1, 5, 6, 9, 11, 15]. Antioxidants such as glutathione, uric acid, and total antioxidant capacity (TAC) are often depleted, further contributing to cellular damage, impaired tissue repair, and increased pain sensitivity [5, 11, 16].

Beyond inflammation and oxidative imbalance, neuroendocrine dysfunction is increasingly recognized as a contributor to the chronicity of TMD pain. Dysregulation of the hypothalamic–pituitary–adrenal (HPA) axis, reflected in altered cortisol rhythms and elevated salivary α-amylase levels, has been associated with increased stress reactivity and pain amplification [5, 13, 17]. Concurrently, reductions in brain-derived neurotrophic factor (BDNF) and nerve growth factor (NGF) may impair neuroplastic responses, contributing to heightened pain perception and limited tissue regeneration [4, 10, 13]. These neuroendocrine alterations appear to be modulated by sex hormones, particularly estrogen, which can influence cytokine production, nociceptor sensitivity, and endogenous opioid pathways. This may partially explain the higher prevalence and severity of TMD in women [6, 10, 18]. Genetic polymorphisms such as those in the *COMT* and *OPRM1* genes have also been linked to sex-specific differences in pain sensitivity and treatment response [18, 19].

Epigenetic regulation further complicates the landscape of chronic TMD. DNA methylation of pain-related genes, including *COMT*, *FKBP5*,* BDNF*,* and TNFα*, has been associated with altered nociceptive thresholds, maladaptive stress responses, and increased vulnerability to pain [20, 23]. Additionally, histone modifications and dysregulation of microRNAs (miRNAs), such as miR-140-5p and miR-21-5p, have been implicated in the maintenance of inflammatory states and degradation of joint cartilage in TMD [6, 22, 24]. These epigenetic alterations can be triggered by environmental stressors, trauma, and hormonal changes, particularly during female reproductive transitions.

This review proposes a translational and clinically oriented framework for understanding chronic TMD pain, grounded in current evidence from molecular biology, neuroendocrinology, and epigenetics. It highlights the diagnostic and prognostic utility of non-invasive biomarkers—particularly those measurables in saliva and blood—and their relevance in advancing precision medicine for orofacial pain. By integrating molecular insights into clinical decision-making, this review offers a roadmap for early identification of chronic pain trajectories, biologically informed risk stratification, and the development of individualized therapeutic strategies tailored to each patient’s molecularprofile.

In addition, this review prioritizes mechanisms supported by evidence in TMD and related TMJ/masticatory muscle pain models, integrating human biomarker studies with preclinical findings where applicable. Mechanisms extensively established in other chronic pain conditions are only discussed when they plausibly intersect with TMD pathophysiology and are explicitly identified as extrapolations that require confirmation in TMD-specific studies.

## Pathophysiological basis of TMD pain chronification

**Fig. 1:**
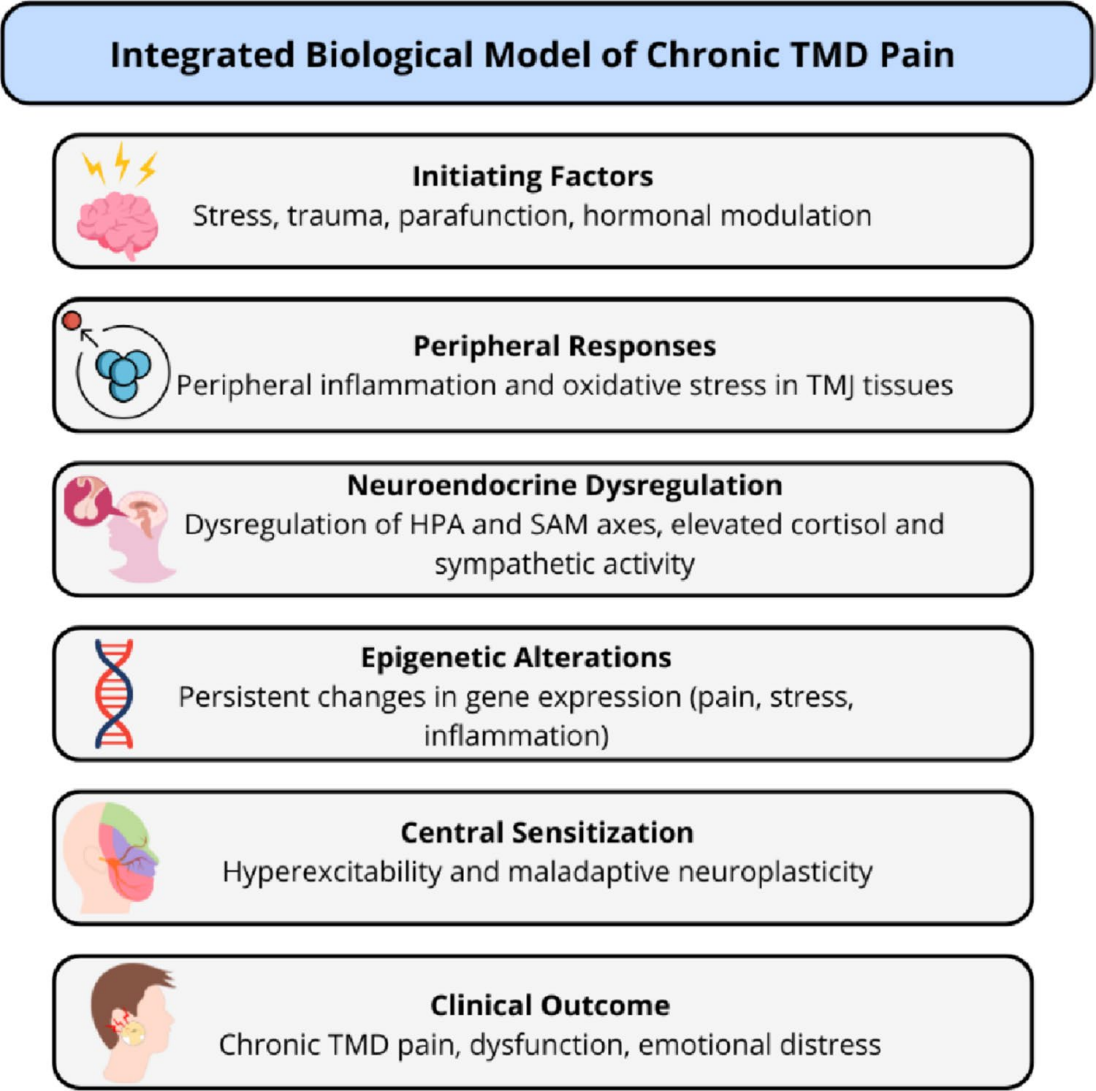
Pathophysiological framework of TMD pain chronification. Schematic representation of the main biological pathways implicated in the transition from acute to chronic TMD pain. Inflammation, oxidative stress, neuroendocrine dysregulation, and neuroplastic maladaptation interact to sustain peripheral and central sensitization. Epigenetic mechanisms further modulate gene expression, contributing to sex-related differences in pain susceptibility and treatment response. Own elaboration (BioRender.com)

The transition from acute to chronic pain in TMD involves a multifaceted process that integrates peripheral and central mechanisms. These include sustained inflammation, oxidative stress, neuroplastic adaptations, and neuroendocrine dysregulation. Understanding these interconnected pathways is crucial for guiding the development of diagnostic biomarkers and targeted therapeutic approaches (Fig. 1).

### Inflammation and peripheral sensitization

Persistent low-grade inflammation is increasingly recognized as a significant factor that plays a central role in the initiation and maintenance of temporomandibular disorder–related pain, extending beyond a passive consequence of tissue injury. Elevated levels of proinflammatory cytokines—particularly interleukin-6 (IL-6), tumor necrosis factor-alpha (TNF-α), and interleukin-1 beta (IL-1β)—have been consistently identified in the saliva, serum, and synovial fluid of individuals with chronic TMD and are associated with increased pain intensity, joint sensitization, and structural degeneration [25, 29, 36]. These cytokines contribute directly to peripheral sensitization by lowering nociceptor activation thresholds and enhancing neuronal excitability, thereby facilitating sustained nociceptive input from the temporomandibular joint and masticatory muscles.

At the molecular level, IL-6, TNF-α, and IL-1β activate intracellular signaling cascades involved in inflammatory amplification and pain persistence, promoting a microenvironment that favors prolonged immune activation and nociceptive signaling. This sustained inflammatory state facilitates communication between peripheral tissues and central nociceptive pathways, contributing to maladaptive plasticity and the maintenance of chronic pain.

In addition to classical cytokines, the adipokine chemerin has been detected at elevated concentrations in temporomandibular joint synovial fluid, where it promotes leukocyte chemotaxis and correlates positively with prostaglandin E2 levels and clinical pain severity [25]. Conversely, reduced circulating levels of cytokines involved in immune regulation, such as CCL4, IL-20, and TWEAK, have been associated with increased susceptibility to TMD, suggesting a systemic imbalance between proinflammatory and regulatory immune mechanisms [27]. Together, these findings indicate that inflammatory mediators not only initiate nociceptive signaling but actively participate in the perpetuation and chronification of TMD-related pain through sustained peripheral sensitization.

### Oxidative stress and redox imbalance

Oxidative stress appears to be a notable contributor to the pathogenesis and chronification of TMD pain. It results from an excess of reactive oxygen species (ROS) and insufficient antioxidant defense mechanisms. Elevated salivary and serum levels of oxidative biomarkers—including malondialdehyde (MDA), 8-hydroxydeoxyguanosine (8-OHdG), and total lipid hydroperoxides—have been consistently associated with pain intensity and emotional distress in TMD patients [30, 34]. Concurrently, reductions in antioxidant markers such as glutathione, uric acid, and total antioxidant capacity (TAC) have been observed, particularly in patients with functional limitations or psychosomatic symptoms [30, 31, 35]. These findings suggest that oxidative imbalance exacerbates inflammatory signaling and contributes to sustained peripheral and central sensitization.

## Central sensitization and neuroplasticity

Recurrent orofacial pain episodes can induce plastic changes in the central nervous system, leading to central sensitization, a state of hyperexcitability that amplifies pain perception. Neuroinflammation and oxidative damage within central pathways may promote aberrant synaptic activity even after resolution of the initial peripheral insult. In TMD-related pain, alterations in neurotrophic factors such as brain-derived neurotrophic factor (BDNF) and nerve growth factor (NGF) have been reported and are thought to modulate nociceptive transmission and neuronal plasticity [33, 37]. Reduced salivary NGF and BDNF have been associated with heightened pain sensitivity and impaired neuronal recovery, [33, 37] whereas increased circulating BDNF reported in some chronic pain phenotypes may reflect maladaptive plasticity rather than effective regeneration [33].

### Neuroendocrine dysregulation and sex differences

Dysregulation of the hypothalamic–pituitary–adrenal (HPA) axis and sympathetic-adrenal-medullary (SAM) system has been implicated in the maintenance of TMD pain. Altered cortisol secretion patterns—such as flattened diurnal rhythms or elevated baseline levels—are associated with increased stress, anxiety, and pain perception [30, 33, 38]. Elevated levels of salivary α-amylase, a marker of sympathetic activation, further support a hyperreactive stress response in TMD patients [30]. These neuroendocrine changes may contribute to emotional dysregulation and amplify nociceptive processing. Significantly, female sex hormones, especially estrogen, influence inflammatory cytokine production and endogenous opioid activity, potentially contributing to the higher prevalence and severity of TMD pain in women [29, 39] Polymorphisms in genes such as *COMT* and *OPRM1*, which regulate catecholamine metabolism and opioid receptor function, have also been linked to sex-specific pain responses in TMD [39, 40].

## Epigenetic regulation of pain in TMD

**Fig. 2:**
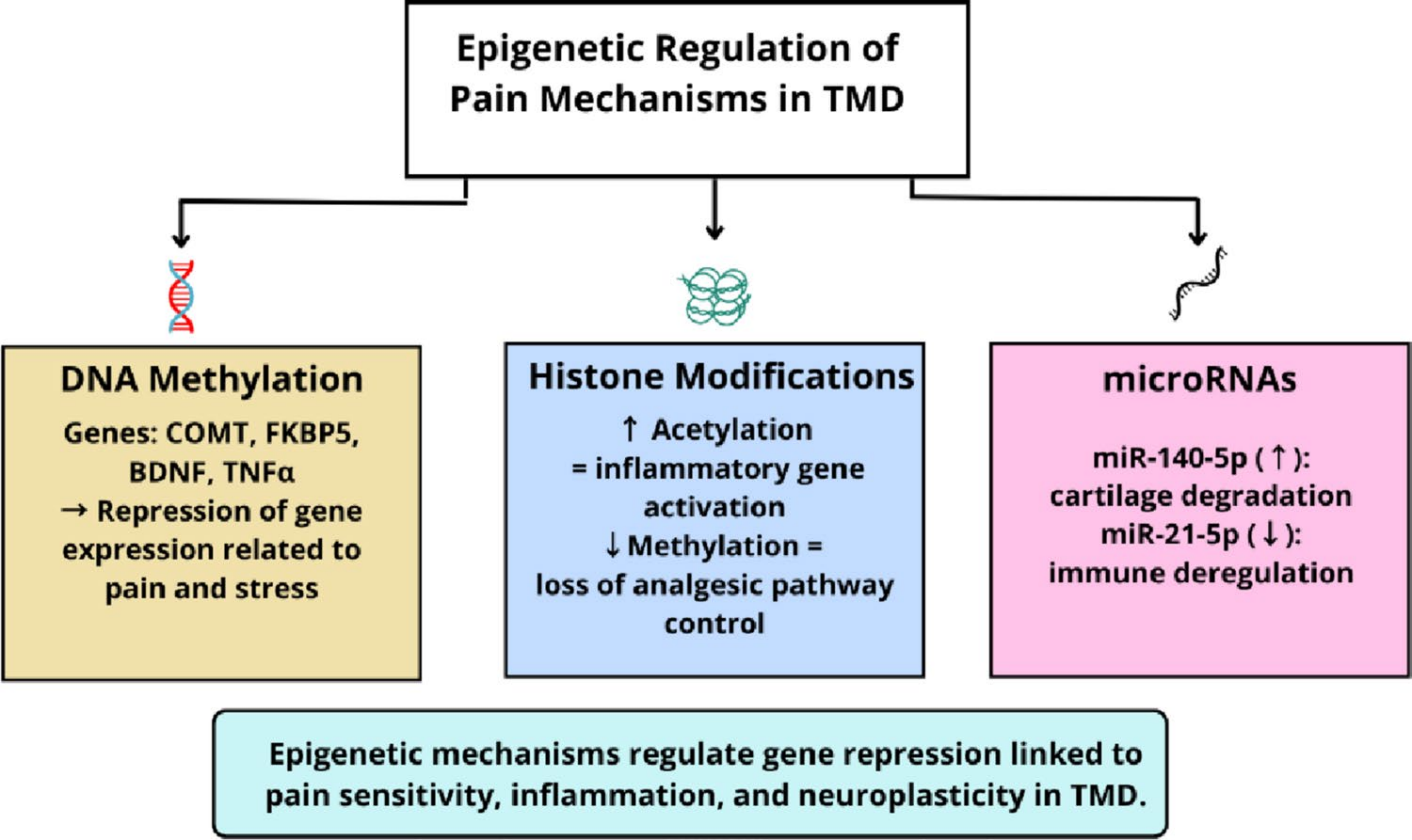
Epigenetic mechanisms involved in TMD-related pain chronification. Overview of the primary epigenetic alterations associated with TMD, including DNA methylation, histone modifications, and microRNA dysregulation. Representative examples (e.g., *COMT*, BDNF, FKBP5, TNFα, miR-140-5p, and miR-21-5p) are highlighted, along with their biological roles and implications for neuroplasticity, inflammation, and stress regulation. Own elaboration (BioRender.com)

In recent years, epigenetic mechanisms have gained prominence as potential regulators of the pathophysiology of chronic pain, including TMD. These processes act as a biological interface between genetic predisposition and environmental influences such as stress, trauma, hormonal fluctuations, and inflammatory states—factors known to be highly relevant in TMD populations. Unlike genetic mutations, epigenetic modifications are reversible and do not alter the DNA sequence; instead, they influence how genes are expressed over time. Among the primary epigenetic mechanisms implicated in pain modulation are DNA methylation, histone modifications, and non-coding RNAs, particularly microRNAs (miRNAs) (Fig. 2). Together, these processes orchestrate transcriptional programs that can promote or inhibit the persistence of nociceptive signaling.

### DNA methylation and transcriptional gene silencing in TMD

DNA methylation typically occurs at CpG dinucleotides and often results in gene silencing when present in promoter regions. In TMD, altered methylation patterns have been identified in several genes relevant to pain. One of the most extensively studied is *COMT* (catechol-O-methyltransferase), which plays a key role in the degradation of catecholamines such as dopamine and norepinephrine. Hypermethylation of *COMT* is associated with decreased enzymatic activity, resulting in increased pain sensitivity and heightened stress responsiveness [41, 42]. Similarly, methylation changes in BDNF (brain-derived neurotrophic factor) have been observed in chronic pain conditions, potentially reducing BDNF expression and impairing neuroplasticity—an essential mechanism in both the maintenance and resolution of nociceptive input [43].

The FKBP5 gene, which encodes a co-chaperone involved in regulating the glucocorticoid receptor, is another epigenetic target relevant to TMD. Methylation at FKBP5 regulatory sites has been linked to prolonged stress exposure, dysregulation of the hypothalamic–pituitary–adrenal (HPA) axis, and increased vulnerability to chronic pain phenotypes [44]. In parallel, the TNFα gene, which encodes a central inflammatory cytokine, has shown promoter methylation patterns consistent with altered immune signaling in TMJ inflammation and chronic orofacial pain [45, 46]. These findings suggest that DNA methylation may not only reflect disease state but also offer predictive value regarding an individual’s susceptibility to persistent TMD symptoms.

### Histone modification and chromatin remodeling

Although TMD-specific studies on histone marks remain scarce, available evidence from chronic orofacial pain and broader chronic pain models suggests that histone acetylation may facilitate transcription of proinflammatory/pronociceptive genes, whereas altered methylation patterns may impair inhibitory pathways [47]. These mechanisms represent plausible contributors to TMD pain chronification but require direct validation in well-phenotyped TMD cohorts.

### MicroRNAs as post-transcriptional regulators

MicroRNAs (miRNAs) are small non-coding RNA molecules that regulate gene expression at the post-transcriptional level by binding to complementary sequences in target mRNAs, leading to mRNA degradation or translational repression. Dysregulation of specific miRNAs has been implicated in the pathogenesis of TMD by promoting chronic inflammation, cartilage degradation, and abnormal nociceptive signaling. For instance, miR-140-5p, which is involved in cartilage homeostasis, has been found upregulated in TMD and contributes to extracellular matrix breakdown and joint dysfunction [48]. In contrast, miR-21-5p, known for its anti-inflammatory and immune-regulatory roles, is often downregulated in chronic pain conditions, suggesting loss of compensatory mechanisms that typically modulate inflammatory cascades [49].

Importantly, several of these miRNAs are detectable in saliva and plasma, making them promising non-invasive biomarkers for diagnosis and treatment monitoring. The dynamic nature of miRNA expression—sensitive to behavioral, hormonal, and pharmacological modulation—also makes them attractive therapeutic targets. Therapies aimed at normalizing miRNA levels, such as miRNA mimics or antagomirs, are currently under investigation in other chronic pain models and could be translated to TMD care.

Table 1 provides an integrative overview of the principal epigenetic alterations implicated in TMD. DNA methylation of genes such as *COMT*, *BDNF*, *FKBP5*, and *TNFα* has been associated with altered stress reactivity, dysregulated neuroplasticity, and increased inflammatory signaling mechanisms that contribute directly to pain chronification. Histone modifications, though less extensively studied in TMD, suggest an epigenomic environment conducive to sustained nociception and inflammatory gene activation. In parallel, microRNAs such as miR-140-5p and miR-21-5p regulate key post-transcriptional pathways involved in maintaining joint integrity and modulating the immune response. Notably, these epigenetic changes are responsive to environmental factors such as psychosocial stress and hormonal fluctuations, and many of their signatures can be measured non-invasively, reinforcing their potential utility as diagnostic and prognostic biomarkers. The inclusion of these markers into clinical workflows may enable earlier identification of at-risk individuals and guide biologically informed personalized interventions in TMD management.

**Table 1:** Epigenetic mechanisms and molecular targets associated with pain chronification in TMD

Epigenetic mechanism	Target gene / Molecule	Biological role	Effect in TMD	Clinical implication	Reference(s)
DNA Methylation	*COMT*	Catecholamine degradation and pain sensitivity	Hypermethylation associated with increased pain perception and reduced stress resilience	Potential biomarker for stress-related pain sensitivity	[41, 42]
DNA Methylation	*BDNF*	Neuroplasticity and neuronal survival	Methylation may reduce neuroplastic response, promoting pain chronification	Predictive of maladaptive plasticity in chronic pain patients	[43]
DNA Methylation	*FKBP5*	Stress regulation and HPA axis modulation	Linked to heightened stress reactivity and vulnerability to chronic pain	Marker of psychosocial stress influence on chronicity	[44]
DNA Methylation	*TNFα*	Inflammatory cytokine production	Associated with elevated inflammation and joint degeneration	Candidate for inflammation-based risk stratification	[45, 46]
Histone Modification	Histone Acetylation	Facilitates transcription of pro-inflammatory genes	Linked to upregulation of inflammatory genes and pain persistence	Possible therapeutic target for anti-inflammatory epigenetic drugs	[47]
Histone Modification	Histone Methylation	May repress anti-nociceptive gene expression	Reduction may dysregulate pain inhibition mechanisms	Suggests utility for restoring gene repression in therapy	[47]
MicroRNA (miRNA)	miR-140-5p	Cartilage integrity and extracellular matrix regulation	Upregulated; contributes to cartilage degradation and joint remodeling	Non-invasive biomarker for cartilage pathology	[48]
MicroRNA (miRNA)	miR-21-5p	Immunomodulation and anti-inflammatory signaling	Downregulated in chronic pain; loss of immune regulatory control	Biomarker of treatment response in inflammation-modulating therapies	[49]

This table presents key epigenetic pathways involved in the modulation of TMD pain. It includes DNA methylation, histone modifications, and microRNAs, highlighting their target genes or molecules, biological roles, effects observed in TMD, and potential clinical applications. The selected targets are based on their relevance to stress reactivity, inflammatory regulation, neuroplasticity, and joint degeneration. References correspond to studies cited throughout the text

### Clinical implications of epigenetic findings in TMD

The integration of epigenetic insights into clinical management of TMD holds significant promise for advancing personalized medicine. Because epigenetic markers are modifiable, they offer the potential for targeted interventions that not only alleviate symptoms but also address underlying biological vulnerability. Behavioral strategies such as stress-reduction techniques (e.g., mindfulness, cognitive-behavioral therapy) and pharmacological agents with epigenetic activity (e.g., histone deacetylase inhibitors) may be developed to reverse maladaptive gene expression profiles in TMD patients. Furthermore, combining epigenetic markers with clinical and psychosocial data may improve diagnostic accuracy, risk stratification, and the prediction of treatment response, ultimately enhancing therapeutic outcomes.

## Neuroendocrine modulation and sex-related differences

**Fig. 3:**
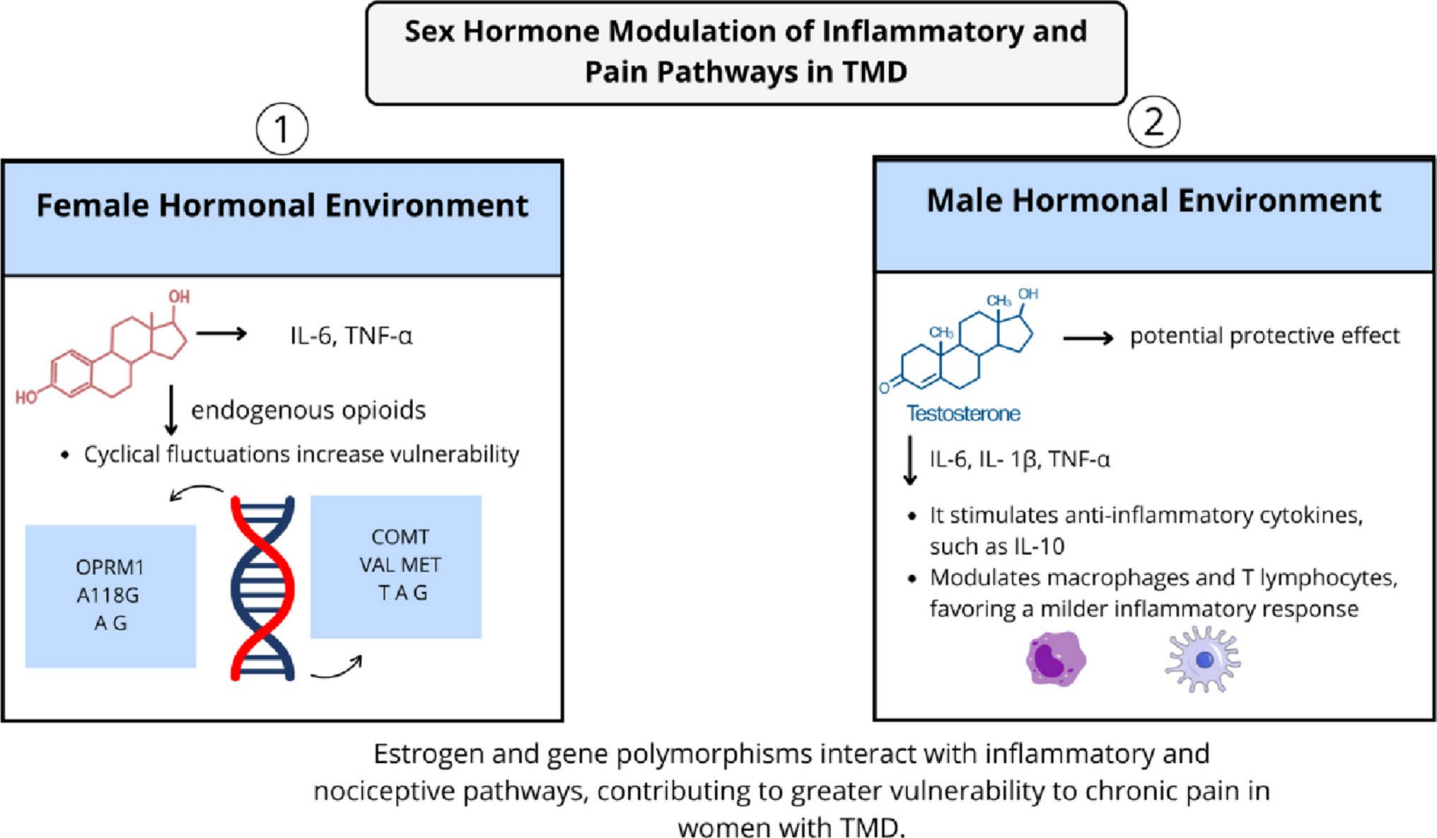
Neuroendocrine modulation and sex-related differences in TMD. Conceptual diagram of HPA and SAM dysregulation in chronic TMD: altered cortisol rhythms and elevated salivary α-amylase reflect heightened stress reactivity; reduced salivary BDNF/NGF indicates impaired neuroplasticity recovery. Overlaid are sex-hormone effects—estrogen-driven immune and nociceptor sensitization versus potentially protective roles of progesterone/testosterone—and gene–hormone interactions (e.g., *COMT*, *OPRM1*) shaping pain susceptibility and treatment response. The pathways converge on peripheral and central sensitization. Own elaboration (BioRender.com)

The neuroendocrine system plays a pivotal role in modulating pain perception and emotional reactivity, particularly in stress-sensitive conditions such as TMD. The hypothalamic–pituitary–adrenal (HPA) axis and the sympathetic-adrenal-medullary (SAM) system are central to the body’s physiological response to stress and pain (Fig. 3). Dysregulation of these systems has been implicated in the pathophysiology of chronic TMD, influencing both the sensory and affective dimensions of pain. Moreover, the modulation of these systems by sex hormones contributes to well-established sex-related differences in TMD prevalence, severity, and treatment outcomes.

### HPA axis dysregulation and pain amplification

Chronic stress exposure and persistent pain stimuli can lead to dysregulation of the HPA axis, characterized by abnormal secretion of cortisol and impaired feedback inhibition. In individuals with TMD, altered cortisol profiles—such as flattened diurnal rhythms or elevated baseline levels—have been consistently associated with increased pain intensity, psychological distress, and impaired coping mechanisms [50, 51]. Salivary cortisol, a reliable non-invasive biomarker of HPA axis activity, has been shown to correlate positively with anxiety, somatization, and functional limitations in TMD patients [50, 52].

Alpha-amylase, an enzyme secreted in response to sympathetic activation, also reflects neuroendocrine stress reactivity. Elevated salivary α-amylase levels have been detected in individuals with TMD and are thought to represent an upregulated SAM system, further contributing to autonomic imbalance and heightened pain sensitivity [50]. These neuroendocrine shifts may promote central sensitization by reducing descending inhibitory control and enhancing excitatory neurotransmission in central nociceptive pathways.

### Neurotransmitters and neuropeptides in pain modulation

Neurotransmitters such as serotonin and dopamine modulate affective processing, motivation, and descending pain control, helping to explain the frequent comorbidity of chronic TMD with anxiety and depressive symptoms [53]. In addition, neuropeptide-related plasticity pathways, including those involving BDNF and NGF have been implicated in TMD-related pain and are discussed in detail in Sect. 1.3 [51, 54].

### Sex hormones and differential pain susceptibility

Sex-related differences in TMD are well documented, with women experiencing higher rates and severity of chronic pain compared to men. This disparity is partially attributable to the modulatory effects of sex hormones—particularly estrogen—on immune, neuroendocrine, and nociceptive systems. Estrogen enhances the expression of proinflammatory cytokines (e.g., IL-6, TNF-α), increases the excitability of nociceptive neurons, and modulates the endogenous opioid system, thereby intensifying pain responses during specific hormonal phases [55, 56].

**Fig. 4:**
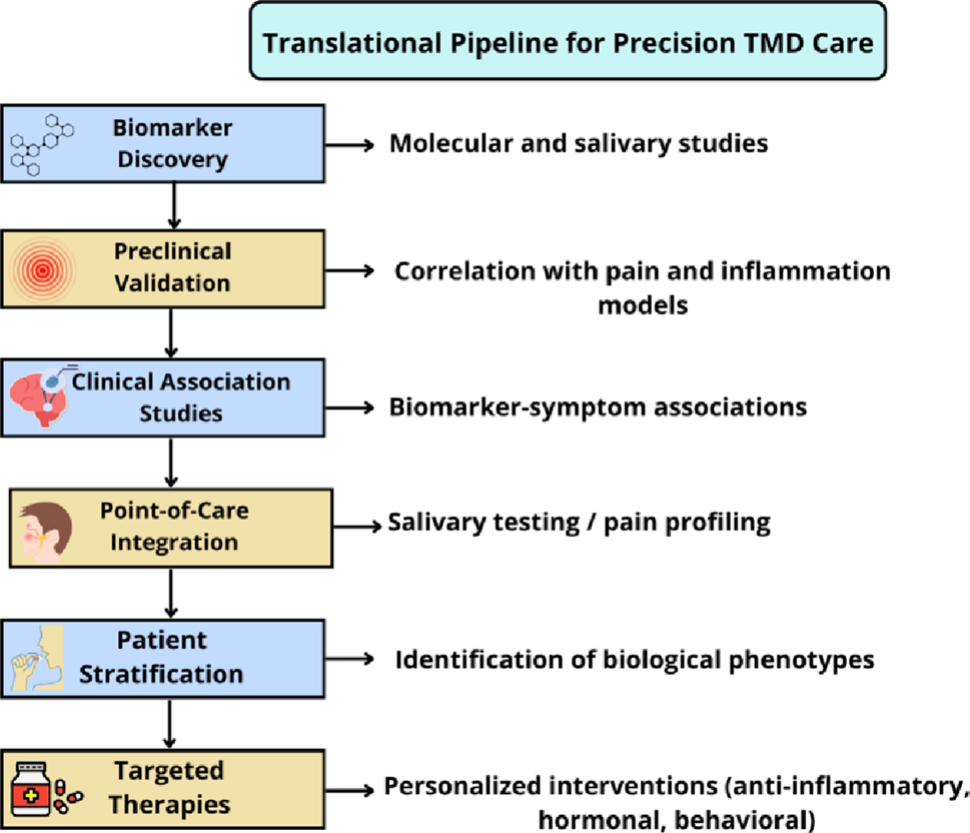
Translational integration of salivary and blood biomarkers for TMD precision care. Illustration of the proposed biomarker-based clinical model. Non-invasive biomarkers measurable in saliva and serum (cytokines, oxidative stress markers, cortisol, BDNF, NGF, and miRNAs) are mapped to their biological pathways (inflammatory, oxidative, neuroendocrine, neuroplasticity, epigenetic). The figure emphasizes their diagnostic, prognostic, and therapeutic utility in guiding individualized treatment strategies. Own elaboration (BioRender.com)

Progesterone and testosterone, in contrast, have been suggested to exert anti-inflammatory and analgesic effects, though their roles in TMD remain less clearly defined [55] Furthermore, hormonal fluctuations during the menstrual cycle, pregnancy, and menopause may exacerbate pain in susceptible individuals, supporting the need for hormone-informed clinical strategies. Recent evidence also suggests the involvement of genetic polymorphisms in pain-relevant genes, such as *COMT* and *OPRM1*, which may interact with hormonal environments to influence pain thresholds and treatment responses in a sex-dependent manner (Fig. 4) [56, 57].

## Translational perspectives and biomarker-based strategies

The integration of molecular, neuroendocrine, and epigenetic findings into TMD management is a promising research direction, but current evidence largely reflects discovery and associative stages. Therefore, biomarker-based tools should be viewed as investigational, requiring standardized protocols and longitudinal validation before clinical implementation.

### Non-invasive biomarkers for risk stratification

A growing body of evidence supports the use of salivary and serum biomarkers to stratify patients according to their biological pain profiles. Proinflammatory cytokines such as IL-6, TNF-α, and IL-1β, when elevated in saliva or blood, have been correlated with greater pain intensity, joint dysfunction, and psychological distress in TMD populations [58, 61]. Oxidative stress markers, including MDA, 8-OHdG, and lipid hydroperoxides, further distinguish patients with higher functional impairment and psychosomatic comorbidity [59, 62]. Neuroendocrine indicators such as salivary cortisol and α-amylase reflect dysregulated stress responses and may predict vulnerability to pain chronification or treatment failure [63]. In parallel, neuroplasticity-related markers like BDNF and NGF offer insights into regenerative capacity and central sensitization [60, 64]. From a practical standpoint, saliva presents an especially attractive diagnostic medium. It is easy to collect, non-invasive, cost-effective, and suitable for repeated sampling. Moreover, salivary concentrations of several biomarkers—including cytokines, neuropeptides, and miRNAs—have shown strong correlations with serum levels and clinical outcomes, supporting their validity for real-time, point-of-care diagnostics in TMD [58, 59, 63].

Table 2 consolidates the main biomarkers discussed in this review, providing a translational perspective on how each marker relates to underlying mechanisms in TMD and its clinical significance. Inflammatory cytokines, such as IL-6 and TNF-α, which are often elevated in saliva and blood, reflect peripheral immune activation and are associated with higher pain intensity and joint dysfunction. Oxidative stress markers, including MDA and 8-OHdG, signal redox imbalance and are linked to psychosomatic manifestations and functional impairment. Neuroendocrine mediators, such as cortisol and α-amylase, provide insight into HPA and sympathetic activation, while neurotrophic factors (BDNF, NGF) reflect the potential for regenerative and neuroplasticity. Additionally, epigenetic signatures—such as *COMT* promoter methylation and altered miRNA profiles (e.g., miR-140-5p, miR-21-5p) —may predict pain sensitivity and treatment responsiveness. This integrative perspective reinforces the value of biological profiling for personalized diagnosis and management in TMD.

**Table 2:** Key salivary and blood biomarkers in TMD: biological pathways and clinical implications

Biomarker	Biological Fluid	Biological Pathway	Clinical Implication	Reference(s)
IL-6	Saliva / Blood	Inflammation	Predicts pain intensity, disease severity	[25, 58, 61]
TNF-α	Saliva / Synovial	Inflammation	Joint degeneration, pain sensitization	[26, 59]
MDA	Saliva / Blood	Oxidative stress	Associated with psychosomatic symptoms	[30, 59]
Cortisol	Saliva	Neuroendocrine (HPA axis)	Marker of stress, predicts chronicity	[50, 63]
BDNF	Saliva / Blood	Neuroplasticity	Reduced regeneration, chronic pain state	[51, 60]
miR-140-5p	Saliva / Blood	Epigenetic, cartilage turnover	Associated with joint degradation	[48, 67]
*COMT* (methyl)	Epigenetic (DNA)	Catecholamine regulation	Predicts pain sensitivity, stress reactivity	[41, 65]

This table summarizes selected biomarkers with clinical relevance in TMD, based on their origin (biological fluid), associated biological mechanisms (inflammatory, neuroendocrine, oxidative, epigenetic), and potential diagnostic or prognostic applications. References correspond to studies cited throughout the text

### Epigenetic signatures and predictive modeling

Epigenetic modifications, including DNA methylation and microRNA (miRNA) expression, provide additional value for individualized assessment. The methylation status of key genes involved in inflammation, stress regulation, and pain modulation—such as *COMT*, *FKBP5*, and *TNFα*—has been linked to pain sensitivity, emotional reactivity, and chronicity in TMD.^65^–^67^ Likewise, circulating and salivary miRNAs such as miR-140-5p and miR-21-5p provide dynamic snapshots of gene expression activity relevant to cartilage degradation, immune response, and nociceptive transmission [67, 68].

By combining epigenetic markers with clinical, psychological, and neuroendocrine data, clinicians can generate individualized risk profiles that predict susceptibility to chronic pain and guide preventive interventions. These multilayered assessments also hold potential for use in early screening, particularly in populations at high risk for TMD (e.g., adolescents, individuals exposed to trauma or high stress).

### Clinical utility and future integration

Despite their promise, several challenges remain in translating biomarker research into routine TMD care. These include the need for standardized collection protocols, large-scale validation in diverse populations, integration with electronic health systems, and cost-effectiveness analyses. Nevertheless, the implementation of biomarker-informed diagnostics and therapeutics could enhance the personalization of TMD management.

In the longer term, and contingent upon rigorous validation, a clinically integrated biomarker panel could support risk stratification and treatment matching (Fig. 4). This will require (i) standardized sampling/assay procedures, (ii) reproducible thresholds and reference ranges, (iii) longitudinal prediction models, and (iv) clinical-effectiveness and cost-effectiveness studies.

Despite the promising findings discussed, several limitations of the current literature must be acknowledged. First, most studies are cross-sectional, yielding primarily associative data that preclude definitive conclusions regarding causality in TMD chronification. Second, many molecular and epigenetic studies are limited by small sample sizes, which may affect the generalizability and robustness of the findings. Finally, the significant clinical heterogeneity of TMD phenotypes—ranging from localized myofascial pain to complex joint degeneration and systemic sensitivity—often complicates the identification of universal biomarkers. Future research should prioritize large-scale, longitudinal cohorts with rigorous phenotyping to validate these biological signatures and confirm their clinical utility.

### Toward a precision TMD care paradigm

Ultimately, the convergence of biomarker science, digital health tools, and machine learning may enable the development of clinical decision-support systems that assist practitioners in diagnosing, monitoring, and tailoring treatment for TMD. Such systems would move beyond the “one-size-fits-all” model and adopt biologically informed precision care, thereby improving outcomes and reducing the burden of chronic orofacial pain. Continued investment in longitudinal cohort studies, translational trials, and interdisciplinary collaboration will be essential to realize this vision.

## Conclusion

Temporomandibular disorders represent a multifactorial group of conditions in which peripheral nociceptive inputs, central sensitization, neuroplastic changes, neuroendocrine dysregulation, and epigenetic mechanisms interact to shape pain persistence and clinical heterogeneity. Accumulating evidence indicates that molecular mediators such as inflammatory cytokines, neurotrophic factors, stress-related hormones, and epigenetic regulators are associated with pain severity, psychological comorbidities, and functional impairment in individuals with TMD. However, much of the available evidence remains associative and derives from observational human studies and preclinical models, underscoring the need for cautious interpretation. While these biological signatures offer a coherent framework to understand interindividual variability and pain chronification in TMD, their translation into clinical practice is still at an early stage. Most candidate biomarkers have not yet undergone sufficient standardization, longitudinal validation, or threshold definition to support diagnostic or prognostic use. Consequently, biomarker-informed approaches should currently be regarded as investigational tools rather than clinically actionable instruments.

Future progress will depend on integrative and longitudinal research designs that combine well-phenotyped clinical cohorts with standardized biomarker assessment and robust analytical models. Such efforts are essential to determine whether biologically informed stratification can meaningfully improve risk assessment, treatment selection, and patient outcomes beyond existing clinical frameworks. Until these steps are achieved, the clinical management of TMD should continue to rely on established biopsychosocial principles, while molecular and neurobiological findings remain valuable primarily for hypothesis generation and the refinement of future translational strategies.

## Data Availability

Data will be made available upon request to the corresponding author.

## References

[1] Kuć J, Szarejko KD, Maciejczyk M, Dymicka-Piekarska V, Żendzian-Piotrowska M, Zalewska A (2025) Oxidative imbalance as a co-player in jaw functional limitations and biopsychosocial profile in patients with temporomandibular disorder-myofascial pain with referral. Front Neurol 15:1509845. 10.3389/fneur.2024.1509845 39830201 10.3389/fneur.2024.1509845 PMC11739092

[2] Jasim H, Ghafouri B, Gerdle B, Hedenberg-Magnusson B, Ernberg M (2020) Altered levels of salivary and plasma pain related markers in temporomandibular disorders. J Headache Pain 21(1):105. 10.1186/s10194-020-01160-z 32842964 10.1186/s10194-020-01160-z PMC7449051

[3] Shekhar A, Maddheshiya N, Nair V, Rastogi V, Srivastava A, Singh AK (2023) Salivary biomarkers and temporomandibular disorders: A systematic review. Natl J Maxillofac Surg 14(3):354–359. 10.4103/njms.njms_136_22 38273906 10.4103/njms.njms_136_22 PMC10806330

[4] Yaman D, Alpaslan C, Akca G, Avcı E (2020) Correlation of molecular biomarker concentrations between synovial fluid and saliva of the patients with temporomandibular disorders. Clin Oral Investig 24(12):4455–4461. 10.1007/s00784-020-03310-8 32385657 10.1007/s00784-020-03310-8

[5] de Sotillo -Rodríguez, Velly D, Hadley AM, Fricton M (2011) Evidence of oxidative stress in temporomandibular disorders: a pilot study. J Oral Rehabil 38(10):722–728. 10.1111/j.1365-2842.2011.02216.x 21457291 10.1111/j.1365-2842.2011.02216.x PMC3153598

[6] Katsiougiannis S, Mallela VR, Schafer CA, Wong DTW (2017) Serum, Synovial, and Salivary Biomarkers for Orofacial Pain Conditions. In: Goulet JP, Velly A (eds) Orofacial Pain Biomarkers. Springer, Berlin, Heidelberg. 10.1007/978-3-662-53994-1_9.

[7] Ao X, Parisien M, Fillingim RB, Ohrbach R, Slade GD, Diatchenko L, Smith SB (2024) Whole-genome methylation profiling reveals regions associated with painful temporomandibular disorders and active recovery processes. Pain 165(5):1060–1073. 10.1097/j.pain.0000000000003104 38015635 10.1097/j.pain.0000000000003104 PMC11018476

[8] Xiong HY, Wyns A, Campenhout JV, Hendrix J, De Bruyne E, Godderis L, Schabrun S, Nijs J, Polli A (2024) Epigenetic Landscapes of Pain: DNA Methylation Dynamics in Chronic Pain. Int J Mol Sci 25(15):8324. 10.3390/ijms25158324 39125894 10.3390/ijms25158324 PMC11312850

[9] Wang Z, Ma H, Nasir A, Liu S, Li Z, Tao F, Bai Q (2024) TET1-mediated epigenetic regulation of tumor necrosis factor-α in trigeminal ganglia contributes to chronic temporomandibular joint pain. Life Sci 336:122283. 10.1016/j.lfs.2023.122283 37993094 10.1016/j.lfs.2023.122283

[10] Maiarù M, Acton RJ, Woźniak EL, Mein CA, Bell CG, Géranton SM (2023) A DNA methylation signature in the stress driver gene FKBP5 indicates a neuropathic component in chronic pain. Clin Epigenetics 15(1):155. 10.1186/s13148-023-01569-8 37777763 10.1186/s13148-023-01569-8 PMC10543848

[11] Zlendić M, Vrbanović E, Trošelj KG, Tomljanović M, Đerfi KV, Alajbeg IZ (2024) Genetic influence on treatment outcomes in patients with pain-related temporomandibular disorders. J Oral Rehabil 51(8):1542–1554. 10.1111/joor.13730 38725226 10.1111/joor.13730

[12] Zlendić M, Vrbanović Đuričić E, Gall Trošelj K, Tomljanović M, Vuković Đerfi K, Alajbeg IZ (2024) Genetic influences of proinflammatory cytokines on pain severity in patients with temporomandibular disorders. Int J Mol Sci 25(16):8730. 10.3390/ijms25168730 39201416 10.3390/ijms25168730 PMC11354298

[13] Zhang SH, Feng Y, Zhong MM, Xie JH, Xu W (2024) Association between oxidative stress and chronic orofacial pain and potential druggable targets: Evidence from a Mendelian randomization study. J Oral Rehabil 51(6):970–981. 10.1111/joor.13663 38414129 10.1111/joor.13663

[14] Ding A, Yu CY, Jiang F, Wu CY, Zhao J (2025) Association between circulating inflammatory proteins and temporomandibular disorders: insight from a two-sample Mendelian randomization analysis. J Appl Oral Sci 32:e20240112. 10.1590/1678-7757-2024-0112 39813538 10.1590/1678-7757-2024-0112 PMC11756819

[15] Achuthan Pk M, Vallikat Velath KS, Varma Nk A (2025) Biomarkers of Temporomandibular Disorders: A Narrative Review. Cureus 17(9):e93390. 10.7759/cureus.93390 41164068 10.7759/cureus.93390 PMC12560064

[16] Alam MK, Zaman MU, Alqhtani NR, Alqahtani AS, Alqahtani F, Cicciù M, Minervini G (2024) Salivary Biomarkers and Temporomandibular Disorders: A Systematic Review conducted according to PRISMA guidelines and the Cochrane Handbook for Systematic Reviews of Interventions. J Oral Rehabil 51(2):416–426. 10.1111/joor.13589 37731276 10.1111/joor.13589

[17] Kazan D, Baş Akkor B, Aksoy A, Atmaca E (2023) The evaluation of oxidative stress and inflammation markers in serum and saliva of the patients with temporomandibular disorders. Turk J Med Sci 53(6):1690–1696. 10.55730/1300-0144.5737 38813510 10.55730/1300-0144.5737 PMC10760560

[18] Eslami H, Katebi K, Ghaffaripour Saleh S, Mirizadeh L, Hashemi M (2024) The relationship between oxidative stress markers and temporomandibular disorders: A systematic review and meta-analysis. J Res Med Sci 29:33. 10.4103/jrms.jrms_660_23 39239079 10.4103/jrms.jrms_660_23 PMC11376713

[19] de Almeida C, Amenábar JM (2016) Changes in the salivary oxidative status in individuals with temporomandibular disorders and pain. J Oral Biol Craniofac Res 6(Suppl 1):S1–S4. 10.1016/j.jobcr.2016.10.006 27900241 10.1016/j.jobcr.2016.10.006 PMC5122870

[20] Vrbanović E (2020) Salivary oxidative stress markers’ levels in patients with temporomandibular disorders (Doctoral dissertation, University of Zagreb. School of Dental Medicine. Department of Removable Prosthodontics)

[21] Aoun Y, Ejbeh R, Youssef A, Hobeiche J (2025) Salivary biomarkers as potential diagnostic tool for temporomandibular disorders: A comprehensive review. Cranio 43(4):603–612. 10.1080/08869634.2023.2229607 37436115 10.1080/08869634.2023.2229607

[22] Farré-Guasch E, Aliberas JT, Spada NF, de Vries R, Schulten EAJM, Lobbezoo F (2023) The role of inflammatory markers in Temporomandibular Myalgia: A systematic review. Jpn Dent Sci Rev 59:281–288. 10.1016/j.jdsr.2023.08.006 37680612 10.1016/j.jdsr.2023.08.006 PMC10480571

[23] Vrbanović E, Zlendić M, Trošelj KG, Tomljanović M, Vuković Đerfi K, Alajbeg IZ (2023) Association of Oxidative-Stress-Related Gene Polymorphisms with Pain-Related Temporomandibular Disorders and Oral Behavioural Habits. Antioxid (Basel) 12(6):1195. 10.3390/antiox12061195 10.3390/antiox12061195 PMC10295756 37371925

[24] Xu M, Fang L, Xue Q, Zhang X, He Y (2023) The Nrf2 Pathway Alleviates Overloading Force-Induced TMJ Degeneration by Downregulating Oxidative Stress Reactions. J Inflamm Res 16:5601–5612. 10.2147/JIR.S434799 38046402 10.2147/JIR.S434799 PMC10691432

[25] Jasim H, Ernberg M, Carlsson A, Gerdle B, Ghafouri B (2020) Protein signature in saliva of temporomandibular disorders Myalgia. Int J Mol Sci 21(7):2569. 10.3390/ijms21072569 32272779 10.3390/ijms21072569 PMC7177369

[26] Zlendić M, Vrbanović E, Tomljanović M, Gall Trošelj K, Đerfi KV, Alajbeg IZ (2024) Association of oral behaviours and psychological factors with selected genotypes in pain-related TMD. Oral Dis 30(3):1702–1715. 10.1111/odi.14583 37036392 10.1111/odi.14583

[27] Bai G, Ross H, Zhang Y, Lee K, Ro JY (2020) The role of DNA methylation in transcriptional regulation of pro-nociceptive genes in rat trigeminal Ganglia. Epigenet Insights 13:2516865720938677. 10.1177/2516865720938677 32974606 10.1177/2516865720938677 PMC7495519

[28] Dong T, Si H, Li Z, Bai Q, Tao F (2022) Transcriptomic analysis of trigeminal ganglion and spinal trigeminal nucleus caudalis in mice with inflammatory temporomandibular joint pain. J Pain Res 15:1487–1502. 10.2147/JPR.S364887 35633917 10.2147/JPR.S364887 PMC9141904

[29] Liu X, Zhao C, Han Y, Feng R, Cui X, Zhou Y, Li Z, Bai Q (2022) RNA sequencing profiling of mRNAs, long noncoding RNAs, and circular RNAs in Trigeminal Ganglion following Temporomandibular Joint inflammation. Front Cell Dev Biol 10:945793. 10.3389/fcell.2022.945793 36051440 10.3389/fcell.2022.945793 PMC9424726

[30] Shrivastava M, Battaglino R, Ye L (2021) A comprehensive review on biomarkers associated with painful temporomandibular disorders. Int J Oral Sci 13(1):23. 10.1038/s41368-021-00129-1 34326304 10.1038/s41368-021-00129-1 PMC8322104

[31] Zhu Y, Gu L, Wang J, Han J, Gou J, Wu Z (2024) DNA methylation profiling of CpG islands in trigeminal ganglion of rats with orofacial pain induced by experimental tooth movement. BMC Oral Health 24(1):1474. 10.1186/s12903-024-05269-4 39633318 10.1186/s12903-024-05269-4 PMC11619421

[32] Dong T, Si H, Li Z, Bai Q, Tao F (2022) Transcriptomic analysis of trigeminal ganglion and spinal trigeminal nucleus caudalis in mice with inflammatory temporomandibular joint pain. J Pain Res 15:1487–1502. 10.2147/JPR.S364887 35633917 10.2147/JPR.S364887 PMC9141904

[33] Sorenson A, Hresko K, Butcher S, Pierce S, Tramontina V, Leonardi R, Loreto C, Bosio J, Almeida LE (2018) Expression of Interleukin-1 and temporomandibular disorder: Contemporary review of the literature. Cranio 36(4):268–272. 10.1080/08869634.2017.1342890 28629271 10.1080/08869634.2017.1342890

[34] Omidpanah N, Ebrahimi S, Raygani AV, Mozafari H, Rezaei M (2020) Total antioxidant capacity, catalase activity and salivary oxidative parameters in patients with temporomandibular disorders. Front Dent 17(16):1–6. 10.18502/fid.v17i16.4179 33615292 10.18502/fid.v17i16.4179 PMC7883650

[35] Oliveira AM, Litke C, Paldy E, Hagenston AM, Lu J, Kuner R, Bading H, Mauceri D (2019) Epigenetic control of hypersensitivity in chronic inflammatory pain by the de novo DNA methyltransferase Dnmt3a2. Mol Pain 15:1744806919827469. 10.1177/1744806919827469 30638145 10.1177/1744806919827469 PMC6362517

[36] Park YM, Ahn YW, Jeong SH, Ju HM, Jeon HM, Kim KH, Ok SM (2019) Interleukin-8 and matrix metalloprotease 9 as salivary biomarkers of pain in patients with temporomandibular disorder myalgia: A pilot study. J Oral Med Pain 44(4):160–168. 10.14476/jomp.2019.44.4.160

[37] Alstergren P (2017) Molecular Temporomandibular Joint Pain Biomarkers. In J.-P. Goulet & A. M. Velly (Eds.), Orofacial Pain Biomarkers. (pp. 95–105). Springer. 10.1007/978-3-662-53994-1_7

[38] Tarcza–Wierzchowska K, Kałuża J, Chaczyńska–Zdybel A et al (2022) Identification of crucial salivary proteins/genes and pathways involved in pathogenesis of temporomandibular disorders. Open Chem 20(1):1378–1401. 10.1515/chem-2022-0249

[39] Ribeiro-Dasilva MC, Fillingim RB, Wallet SM (2017) Estrogen-Induced Monocytic Response Correlates with TMD Pain: A Case Control Study. J Dent Res 96(3):285–291. 10.1177/0022034516678599 27856968 10.1177/0022034516678599 PMC5298393

[40] Leppilahti JM, Knuutila J, Pesonen P, Vuollo V, Männikkö M, Karjalainen MK, Suominen AL, Sipilä K (2025) Genome-wide association study of temporomandibular disorder-related pain in finnish populations. J Oral Rehabil 52(2):151–159. 10.1111/joor.13883 39482899 10.1111/joor.13883 PMC11740273

[41] Zwiri AM, Ahmad WMAW, Asif JA, Phaik KS, Husein A, Kassim NK, Ab-Ghani Z (2022) A randomized controlled trial evaluating the levels of the biomarkers hs-CRP, IL-6, and IL-8 in patients with temporomandibular disorder treated with LLLT, traditional conservative treatment, and a combination of both. Int J Environ Res Public Health 19(15):8987. 10.3390/ijerph19158987 35897358 10.3390/ijerph19158987 PMC9332699

[42] Fox S, Tiwari L, Farah C (2020) Epigenetics and oral disease. In S. T. Sonis & A. Villa (Eds.), Translational Systems Medicine and Oral Disease. (1st ed., pp. 163–196). Elsevier. 10.1016/B978-0-12-813762-8.00007-4

[43] Raghavan S, Al-Hamed FS, Al-Hadeethi T, Danadneh M, Alhaija ESA (2026) Salivary stress biomarkers in subjects with temporomandibular disorders (TMDs): a meta-analysis. J Oral Rehabil 12. 10.1111/joor.70154 10.1111/joor.70154 41677029

[44] Smith SB, Parisien M, Bair E, Belfer I, Chabot-Doré AJ, Gris P, Khoury S, Tansley S, Torosyan Y, Zaykin DV, Bernhardt O, de Oliveira Serrano P, Gracely RH, Jain D, Järvelin MR, Kaste LM, Kerr KF, Kocher T, Lähdesmäki R, Laniado N, Laurie CC, Laurie CA, Männikkö M, Meloto CB, Nackley AG, Nelson SC, Pesonen P, Ribeiro-Dasilva MC, Rizzatti-Barbosa CM, Sanders AE, Schwahn C, Sipilä K, Sofer T, Teumer A, Mogil JS, Fillingim RB, Greenspan JD, Ohrbach R, Slade GD, Maixner W, Diatchenko L (2019) Genome-wide association reveals contribution of MRAS to painful temporomandibular disorder in males. Pain 160(3):579–591. 10.1097/j.pain.0000000000001438 30431558 10.1097/j.pain.0000000000001438 PMC6377338

[45] Qiu D, Sun S (2024) Causal relationships between immunophenotypes, plasma metabolites, and temporomandibular disorders based on Mendelian randomization. Sci Rep 14(1):22262. 10.1038/s41598-024-73330-x 39333658 10.1038/s41598-024-73330-x PMC11436868

[46] Staniszewski K, Lygre H, Bifulco E, Kvinnsland S, Willassen L, Helgeland E, Berge T, Rosén A (2018) Temporomandibular Disorders Related to Stress and HPA-Axis Regulation. Pain Res Manag 2018:7020751. 10.1155/2018/7020751 29854038 10.1155/2018/7020751 PMC5954859

[47] Luo D, Li X, Tang S, Song F, Li W, Xie G, Liang J, Zhou J (2021) Epigenetic modifications in neuropathic pain. Mol Pain 17:17448069211056767. 10.1177/17448069211056767 34823400 10.1177/17448069211056767 PMC8647219

[48] Vrbanović E, Zlendić M, Trošelj KG, Tomljanović M, Vuković Đerfi K, Alajbeg IZ (2023) Association of oxidative-stress-related gene polymorphisms with pain-related temporomandibular disorders and oral behavioural habits. Antioxid (Basel) 12(6):1195. 10.3390/antiox12061195 10.3390/antiox12061195 PMC10295756 37371925

[49] Aneiros-Guerrero A, Lendinez AM, Palomares AR, Perez-Nevot B, Aguado L, Mayor-Olea A, Ruiz-Galdon M, Reyes-Engel A (2011) Genetic polymorphisms in folate pathway enzymes, DRD4 and GSTM1 are related to temporomandibular disorder. BMC Med Genet 12:75. 10.1186/1471-2350-12-75 21615938 10.1186/1471-2350-12-75 PMC3129576

[50] Meyer MK, Ismail E, Chetty M (2024) Investigating the association between Catechol-O-Methyltransferase gene activity and pain perception in South African patients with different temporomandibular disorders diagnoses. Biomedicines 12(10):2331. 10.3390/biomedicines12102331 39457643 10.3390/biomedicines12102331 PMC11505128

[51] Ismah N, Bachtiar EW, Purwanegara MK, Tanti I, Mardiati E (2024) Evaluation of IL-1β and CRP mRNA expression levels by RT-PCR in postorthodontic treatment patients with temporomandibular joint disorders: a cross-sectional Study. J Int Soc Prev Community Dent 14(2):98–104. 10.4103/jispcd.jispcd_197_23 38827355 10.4103/jispcd.jispcd_197_23 PMC11141896

[52] Pethő G, Kántás B, Horváth Á, Pintér E (2023) The epigenetics of neuropathic pain: a systematic update. Int J Mol Sci 24(24):17143. 10.3390/ijms242417143 38138971 10.3390/ijms242417143 PMC10743356

[53] Deng H, Zhou P, Wang J, Zeng J, Yu C (2025) CircRNA expression profiling of the rat thalamus in temporomandibular joint chronic inflammatory pain. Gene 934:149024. 10.1016/j.gene.2024.149024 39433265 10.1016/j.gene.2024.149024

[54] Zwiri A, Al-Hatamleh MAI, Ahmad W, Ahmed Asif WMA, Khoo J, Husein SP, Ab-Ghani A, Kassim Z (2020) Biomarkers for temporomandibular disorders: current status and future directions. Diagnostics (Basel) 10(5):303. 10.3390/diagnostics10050303 32429070 10.3390/diagnostics10050303 PMC7277983

[55] Yi Y, Zhou X, Xiong X, Wang J (2021) Neuroimmune interactions in painful TMD: Mechanisms and treatment implications. J Leukoc Biol 110(3):553–563. 10.1002/JLB.3MR0621-731RR 34322892 10.1002/JLB.3MR0621-731RR

[56] Almeida LE, Doetzer A, Beck ML (2023) Immunohistochemical markers of temporomandibular disorders: a review of the literature. J Clin Med 12(3):789. 10.3390/jcm12030789 36769438 10.3390/jcm12030789 PMC9917491

[57] Jośko–Ochojska J (2019) Epigenetic understanding of pain mechanisms and modern treatment perspectives. Ból 20(1):45–53. 10.5604/01.3001.0013.4582

[58] Asquini G, Devecchi V, Viscuso D, Bucci R, Michelotti A, Liew BXW, Falla D (2025) An exploratory data-driven approach to classify subgroups of patients with temporomandibular disorders based on pain mechanisms. J Pain 26:104721. 10.1016/j.jpain.2024.104721 39461455 10.1016/j.jpain.2024.104721

[59] Jiang W, Zhang LX, Tan XY, Yu P, Dong M (2023) Inflammation and histone modification in chronic pain. Front Immunol 13:1087648. 10.3389/fimmu.2022.1087648 36713369 10.3389/fimmu.2022.1087648 PMC9880030

[60] Bai Q, Zhou Y, Cui X, Si H, Wu T, Nasir A, Ma H, Xing J, Wang Y, Cheng X, Liu X, Qi S, Li Z, Tang H (2023) Correction: Mitochondria-targeting nanozyme alleviating temporomandibular joint pain by inhibiting the TNFα/NF-κB/NEAT1 pathway. J Mater Chem B 12(1):275–276. 10.1039/d3tb90183a. Erratum for: J Mater Chem B. 2023;12(1):112–121. doi: 10.1039/d3tb00929g 38054383 10.1039/d3tb90183a

[61] Zhang Z, Yuan L, Liu Y, Wang R, Zhang Y, Yang Y, Wei H, Ma J (2023) Integrated cascade nanozyme remodels chondrocyte inflammatory microenvironment in temporomandibular joint osteoarthritis via inhibiting ROS-NF-κB and MAPK Pathways. Adv Healthc Mater 12(10):e2203195. 10.1002/adhm.202203195 36738173 10.1002/adhm.202203195

[62] Niederberger E, Resch E, Parnham MJ, Geisslinger G (2017) Drugging the pain epigenome. Nat Rev Neurol 13(7):434–447. 10.1038/nrneurol.2017.68 28548108 10.1038/nrneurol.2017.68

[63] Sumantri DDD, Naliani S, Lelyana S, Sandra F (2019) Biomarkers of temporomandibular disorders. SONDE 3(1):41–47. 10.28932/SOD.V3I1.1782

[64] Lu G, Du R (2024) Temporomandibular joint disorder: An integrated study of the pathophysiology, neural mechanisms, and therapeutic strategies. Arch Oral Biol 164:106001. 10.1016/j.archoralbio.2024.106001 38749387 10.1016/j.archoralbio.2024.106001

[65] Pavithra M, Muthukrishnan A (2023) Role of oxidative stress as an etiological agent in temporomandibular disorders: a systematic review. J Popul Ther Clin Pharmacol 30(10):154–161. 10.47750/jptcp.2023.30.10.020

[66] Chen H, Comnick C, Norman GJ, Caplan DJ, Xie XJ, Fillingim RB (2023) Triad multisystem phenotype with high risk for developing temporomandibular disorders-characteristics and potential pathophysiology results from the Orofacial pain: prospective evaluation and risk assessment dataset. Pain 164(5):1027–1038. 10.1097/j.pain.0000000000002797 36661844 10.1097/j.pain.0000000000002797

[67] Stenz L, Carré JL, Luthi F, Vuistiner P, Burrus C, Paoloni-Giacobino A, Léger B (2022) Genome-wide epigenomic analyses in patients with nociceptive and neuropathic chronic pain subtypes reveals alterations in methylation of genes involved in the neuro-musculoskeletal system. J Pain 23(2):326–336. 10.1016/j.jpain.2021.09.001 34547430 10.1016/j.jpain.2021.09.001

[68] Louca Jounger S, Christidis N, Svensson P, List T, Ernberg M (2017) Increased levels of intramuscular cytokines in patients with jaw muscle pain. J Headache Pain 18(1):30. 10.1186/s10194-017-0737-y 28243900 10.1186/s10194-017-0737-y PMC5328896

